# Long-Term Compost Amendment Spurs Cellulose Decomposition by Driving Shifts in Fungal Community Composition and Promoting Fungal Diversity and Phylogenetic Relatedness

**DOI:** 10.1128/mbio.00323-22

**Published:** 2022-05-02

**Authors:** Yuncai Miao, Junjie Li, Ye Li, Yuhui Niu, Tiehu He, Deyan Liu, Weixin Ding

**Affiliations:** a State Key Laboratory of Soil and Sustainable Agriculture, Institute of Soil Science, Chinese Academy of Sciences, Nanjing, China; b University of Chinese Academy of Sciences, Beijing, China; CEH—Oxford

**Keywords:** [^13^C]cellulose, fungi, long-term fertilization, RNA, stable isotope probing

## Abstract

Cellulose is the most abundant polysaccharide in plant biomass and an important precursor of soil organic matter formation. Fungi play a key role in carbon cycling dynamics because they tend to decompose recalcitrant materials. Here, we applied [^12^C]cellulose and [^13^C]cellulose to distinguish the effects of application of compost, nitrogen-phosphorus-potassium (NPK) fertilizer, and no fertilizer (control) for 27 years upon cellulose decomposition via RNA-based stable isotope probing (RNA-SIP). The loss ratio of added cellulose C in compost soil was 67.6 to 106.7% higher than in NPK and control soils during their 20-day incubation. *Dothideomycetes* (mainly members of the genus Cryptococcus) dominated cellulose utilization in compost soil, whereas the copiotrophic *Sordariomycetes* were more abundant in NPK and unfertilized soils. Compared with NPK and control soils, compost application increased the diversity of ^13^C-assimilating fungi. The ^13^C-labeled fungal communities in compost soil were more phylogenetically clustered and exhibited greater species relatedness than those in NPK and control soils, perhaps because of stringent filtering of narrow-spectrum organic resources and biological invasion originating from added compost. These changes led to an augmented decomposition capacity of fungal species for cellulose-rich substrates and reduced cellulose C sequestration efficiency. The RNA-SIP technique is more sensitive to responses of fungi to altered soil resource availability than DNA-SIP. Overall, long-term compost application modified fungal community composition and promoted fungal diversity and phylogenetic relatedness, accelerating the decomposition of substrate cellulose in soil. This work also highlights the RNA-SIP technique’s value for comprehensively assessing the contributions of active fungi to the substrate decomposition process.

## INTRODUCTION

Increasing soil organic carbon (SOC) sequestration improves soil fertility and mitigates climate change (1). The input of organic materials such as crop residues, whose biomass is now 3.8 × 10^9^ tons year^−1^ globally (2), offers an effective and promising approach to sequester more SOC (3, 4). Cellulose is the richest component in crop residues (5), but its degradation depends on the concerted action of multiple enzymes, such as endoglucanases, cellobiohydrolases, and β-glucosidase (6). During the decomposition of cellulose, part of cellulose-derived C is mineralized into CO_2_, whereas the other portion can accumulate in soil as microbial necromass and metabolites (7). Fungi are pivotal for cellulose decomposition because they can extend their hyphae to access substrates and produce extracellular enzymes which break down recalcitrant compounds, namely, cellulose (8). Certain fungal taxa, such as *Sordariomycetes*, *Staphylotrichum*, and *Dothideales*, are the main utilizers of cellulose in soils (9, 10). However, a fundamental understanding of how fungal community composition and diversity affect cellulose decomposition is still lacking.

Long-term application of organic fertilizers to soil can shift fungal community composition toward more saprotrophic fungi and higher fungal diversity (11, 12), possibly due to the increased organic substances and colonization by exogenous species from organic amendments (13). Recently, Fang et al. (14) found that an increase in saprotrophic fungal abundance resulted in higher rates of decomposition of leaf litter in forest soil around arbuscular mycorrhizal trees than ectomycorrhizal trees. Earlier, Ling et al. (15) demonstrated that in comparison with chemical fertilizers, organic amendments support stronger functional potential by enhancing the diversity and abundance of functional groups with respect to C-, N- and P-related metabolism. In particular, it has been shown that cocultures of diverse species can break down substrate biomass (i.e., lignocellulose and cellulose) more efficiently than can the same species in monocultures (16, 17). In general, greater microbial diversity entails more complex microbial interactions and effectively promotes soil functioning, such as C decomposition, by producing complementary enzymes acting at different sites of targeted compounds or by enhancing overall enzyme production (18–20). For example, “sugar” fungi, which cannot break down cellulose, are able to use the labile products of cellulose decomposition by cellulolytic fungi, such as cellobiose (21). This contributes to improving the cellulase activities of cellulolytic species by alleviating product inhibition (22), thereby accelerating the substrates’ decomposition.

Nucleic acid-based stable isotope probing (SIP), whereby stable isotopes such as ^13^C derived from labeled substrates are incorporated into microbial nucleic acids followed by high-throughput sequencing, can provide a way to link phylogenetic information of microbes to their functioning (23). The DNA-SIP technique has been widely used to investigate active microbes utilizing organic substances, such as the organic compounds glucose (24), cellulose (10, 25), and lignin (26), as well as some heterogeneous materials, such as straw residues (27) and root (28). However, because DNA has a long residence time in soil, any relic DNA, including extracellular DNA and nondecomposed DNA from dead cells, may obscure the real changes in metabolically active microbial communities (29). In contrast, RNA-SIP has higher sensitivity than DNA-SIP due to the faster turnover and isotopic incorporation of RNA than DNA (30); hence, it is useful for identifying microbial communities that are actively involved in ecological processes at the temporal scale of sampling. However, the instability of RNA renders this technique more challenging for assessing specific functions of the active microbial community.

In this study, soils sampled from the plots of three treatments in a long-term (27-year) fertilization field experiment were incubated with [^12^C]cellulose and [^13^C]cellulose. ^13^C RNA-SIP with subsequent high-throughput sequencing was used to characterize the soil fungal communities during cellulose decomposition. The objectives were 3-fold: (i) to identify ^13^C-assimilating fungal communities and determine their impact on cellulose decomposition, (ii) to evaluate the influence on cellulose-using fungi of different fertilizers’ application, and (iii) to compare differences in the response of fungal species to cellulose amendment as determined by RNA-SIP and DNA-SIP techniques. We hypothesized that long-term compost application alters fungal community composition, thereby stimulating cellulose decomposition and turnover to soil organic matter.

## RESULTS

### Soil properties and cellulose decomposition rate.

Compared with NPK addition and no addition (control), adding compost significantly (*P < *0.05) increased the organic C, total N, available P, and available K of soil but did not affect its C/N ratio or pH (Fig. S1). During the 20-day incubation, 38% of cellulose-derived ^13^C was retained in compost soil, which was significantly (*P < *0.05) less than that retained in NPK (63%) and control (70%) soils (Fig. S2).

10.1128/mbio.00323-22.1 FIG S1 Basal soil characteristics after 27 years of fertilization. The whiskers denote standard errors of the means (*n *= 3). Different letters indicate significant differences among the fertilization treatments, at a *P* value of <0.05. Download FIG S1, DOCX file, 0.2 MB. Copyright © 2022 Miao et al. 2022 Miao et al. https://creativecommons.org/licenses/by/4.0/ This content is distributed under the terms of the Creative Commons Attribution 4.0 International license.

10.1128/mbio.00323-22.2 FIG S2 Proportion (percent) of cellulose ^13^C remaining in soil at 20 days of incubation. The whiskers denote standard errors of the means (*n *= 3). Different letters indicate significant differences among the fertilization treatments, at a *P* value of <0.05. Download FIG S2, DOCX file, 0.08 MB. Copyright © 2022 Miao et al. 2022 Miao et al. https://creativecommons.org/licenses/by/4.0/ This content is distributed under the terms of the Creative Commons Attribution 4.0 International license.

### ^13^C-assimilating fungal community composition and diversity.

Fungal RNA from the [^13^C]cellulose microcosms was more abundant in the heavy fractions (buoyant densities of 1.790 to 1.820 g mL^−1^), whereas that from [^12^C]cellulose microcosms was enriched in the light fractions (buoyant densities of 1.767 to 1.784 g mL^−1^) (Fig. S3). We selected fungal RNA in the heavy fractions from both [^12^C]cellulose and [^13^C]cellulose microcosms for the high-throughput sequencing analysis. Principal-coordinate analysis (PCoA) (Fig. S4) revealed a different fungal community composition in the heavy fractions of [^13^C]cellulose microcosms versus [^12^C]cellulose microcosms. Here, fungal microorganisms in the heavy fractions from [^13^C]cellulose microcosms were defined as ^13^C-assimilating fungal taxa.

10.1128/mbio.00323-22.3 FIG S3 Relative abundances of fungal ITS RNA transcripts, based on the CsTFA buoyant density, at 20 days of incubation with [^12^C]cellulose (blue) and [^13^C]cellulose (red). The amounts of ITS-RNA transcripts were determined by qPCR, for which cDNA served as the template. Download FIG S3, DOCX file, 0.2 MB. Copyright © 2022 Miao et al. 2022 Miao et al. https://creativecommons.org/licenses/by/4.0/ This content is distributed under the terms of the Creative Commons Attribution 4.0 International license.

10.1128/mbio.00323-22.4 FIG S4 Principal-coordinate analysis (PCoA) of fungal communities in the heavy fractions across the fertilization treatments receiving the [^12^C]cellulose and [^13^C]cellulose additions, based on Bray-Curtis distances at the OTU level, at 20 days of incubation. Download FIG S4, DOCX file, 0.2 MB. Copyright © 2022 Miao et al. 2022 Miao et al. https://creativecommons.org/licenses/by/4.0/ This content is distributed under the terms of the Creative Commons Attribution 4.0 International license.

**FIG 1:**
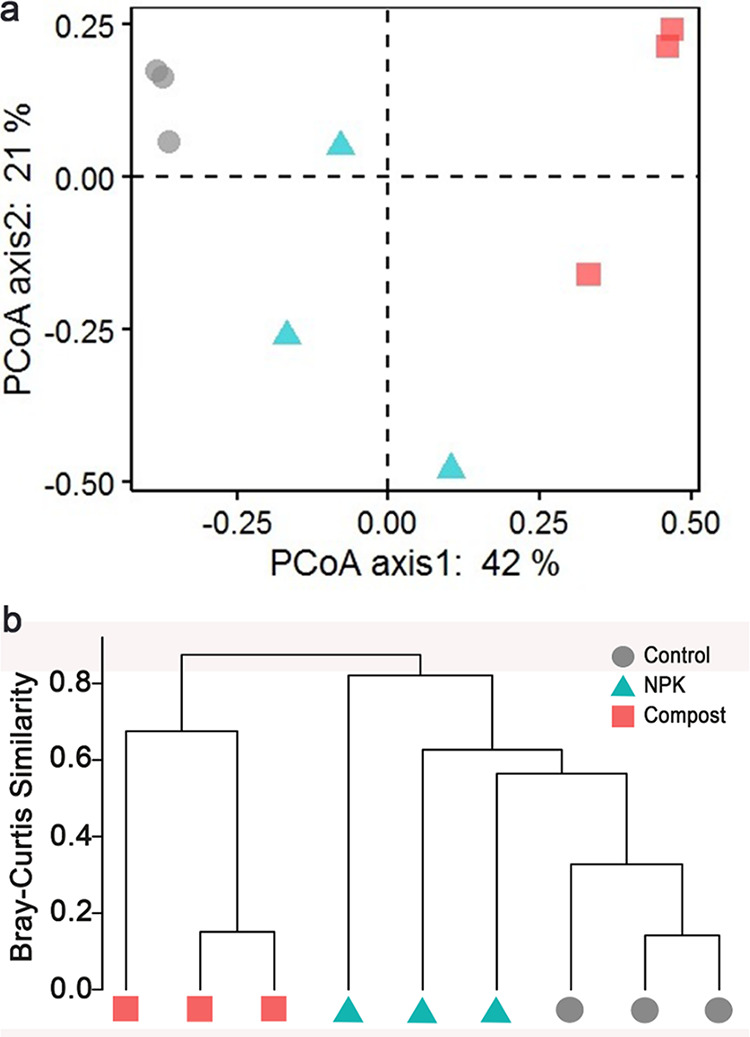
Principal-coordinate analysis (PCoA) and hierarchical clustering analysis of ^13^C-assimilating fungal communities with OTUs classified at 97% sequence similarity, based on Bray-Curtis distances.

**FIG 2:**
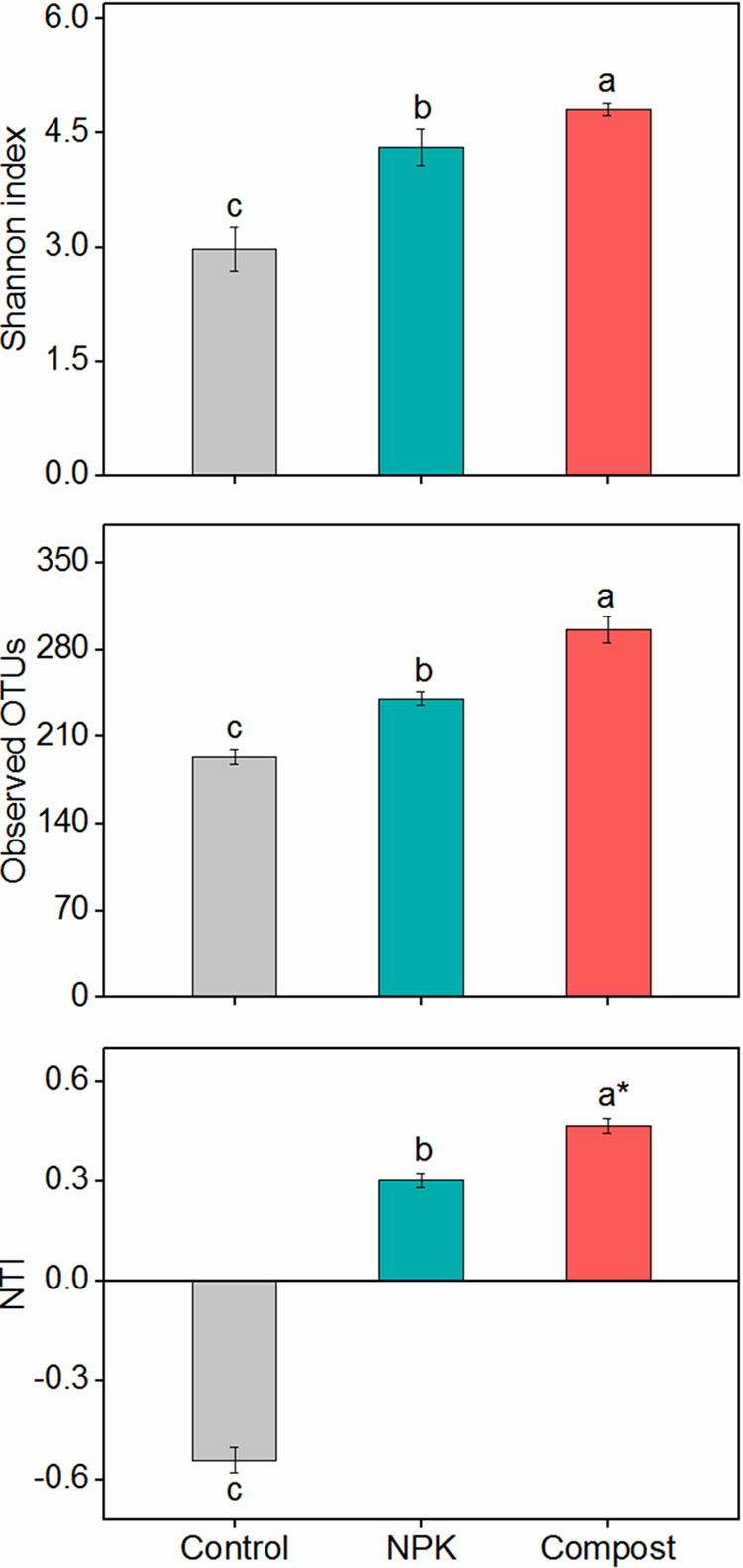
Shannon diversity, observed OTUs, and nearest-taxon indexes (NTI) of ^13^C-assimilating fungi in the soils undergoing long-term fertilization. The whiskers denote standard errors of the means (*n *= 3). Different letters and the asterisk indicate significant differences (*P < *0.05) among the three fertilization treatments and between NTI values and zero, respectively.

Long-term compost amendment altered the ^13^C-labeled fungal community structure (Fig. 1a). The hierarchical clustering analysis showed that fungal communities in compost soil were significantly distinguished from those in NPK and control soils (Fig. 1b). Compost soil increased the diversity of ^13^C-assimilating fungi compared with that of NPK and unfertilized soils (Fig. 2). The nearest-taxon index (NTI) in compost soil reached 0.47, a value significantly greater than zero (*P < *0.05), whereas for the NPK (0.30) and control (−0.54) soils, neither value differed significantly from zero. These results indicated that the ^13^C-labeled fungal communities in compost soil were phylogenetically clustered, in contrast with the expected random clustering and dispersion of fungal microorganisms in NPK and control soils, respectively.

**FIG 3:**
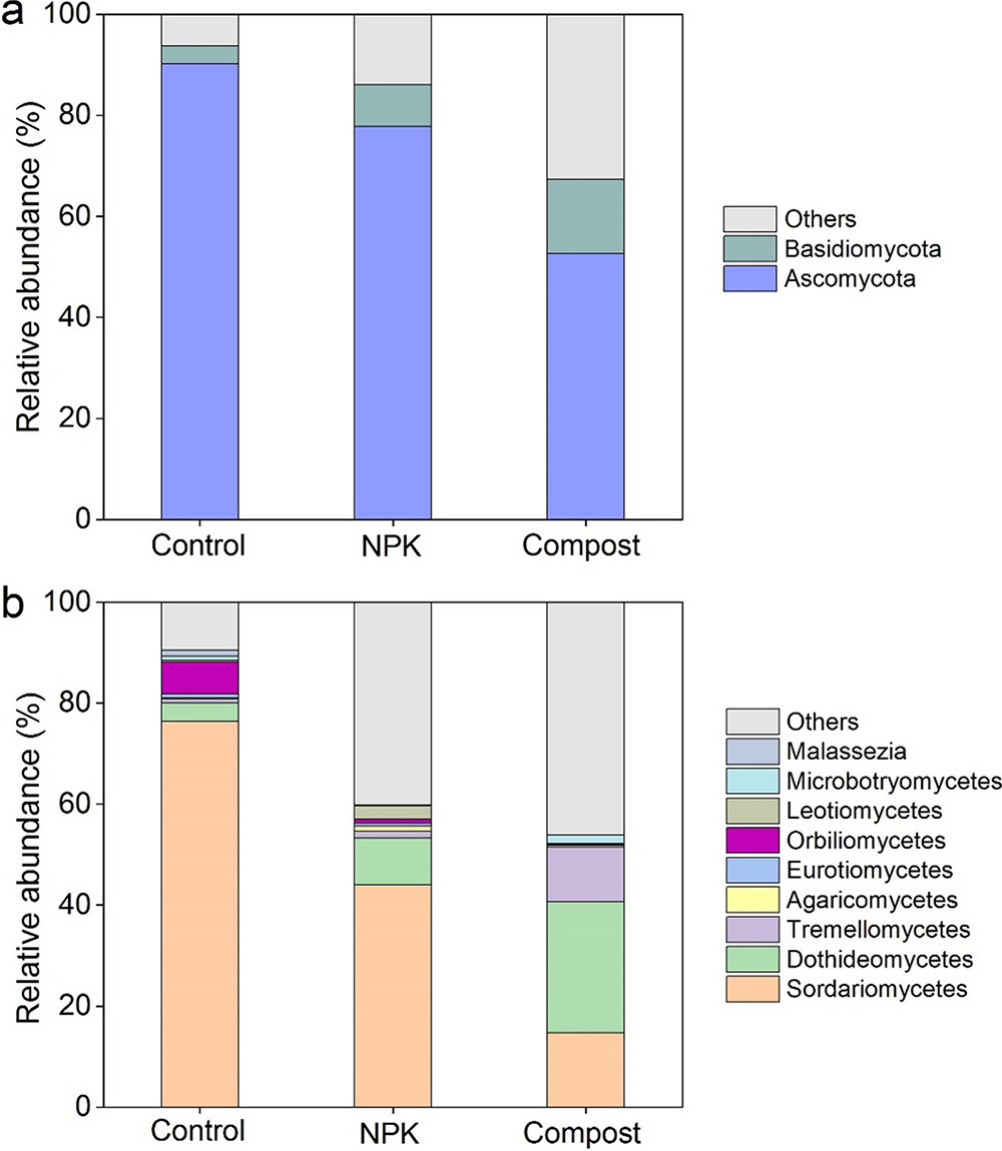
Relative abundances of major phyla (a) and classes or genera (b) among members of ^13^C-assimilating fungal communities (>1%) occurring in soils undergoing long-term fertilization. The OTUs annotated as class *incertae sedis* were all assigned to the genus *Malassezia*.

**FIG 4:**
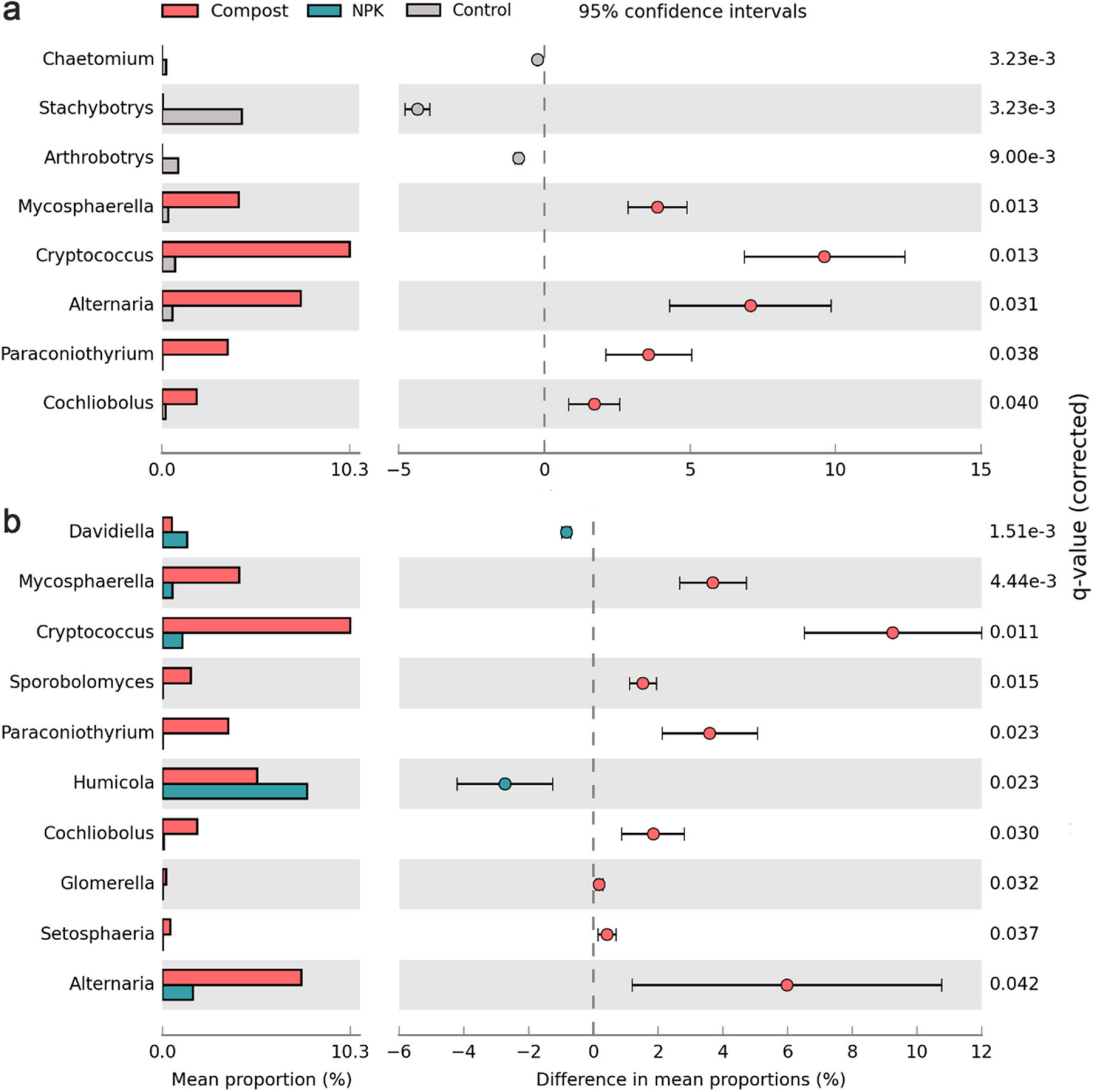
STAMP analysis exhibiting the differentially abundant genera among members of ^13^C-assimilating fungal populations in compost treatment compared with NPK and control treatments.

Cellulose was mainly utilized by *Ascomycetes* across the various fertilization treatments (Fig. 3a). The compost treatment yielded a lower relative abundance of *Ascomycetes* (53%) than NPK treatment (78%) and control treatment (90%). In stark contrast, *Basidiomycota* increased from 3.5 to 8.2% in NPK and control soils to 15% in compost soil. At the class level, *Dothideomycetes* (26%) were the most abundant in compost soil, followed by *Sordariomycetes* (15%) and then *Tremellomycetes* (11%) (Fig. 3b). This contrasts with *Sordariomycetes* being predominantly responsible for cellulose utilization in NPK and control soils, accounting for 44% and 76%, respectively, of their total fungal species. To better understand the effect of compost application on ^13^C-labeled fungal community composition, significantly different biomarkers at the genus level in compost treatment were analyzed (Fig. 4). Compared with NPK and control soils, the genus Cryptococcus was markedly (*P < *0.05) enriched in compost soil, with the highest relative abundance among these biomarker taxa. Additionally, *Alternaria*, *Mycosphaerella*, *Paraconiothyrium*, and *Cochliobolus*, which are all affiliated with the class *Dothideomycetes*, were also increased in compost treatment.

### Association of cellulose decomposition rate with fungal communities.

**FIG 5:**
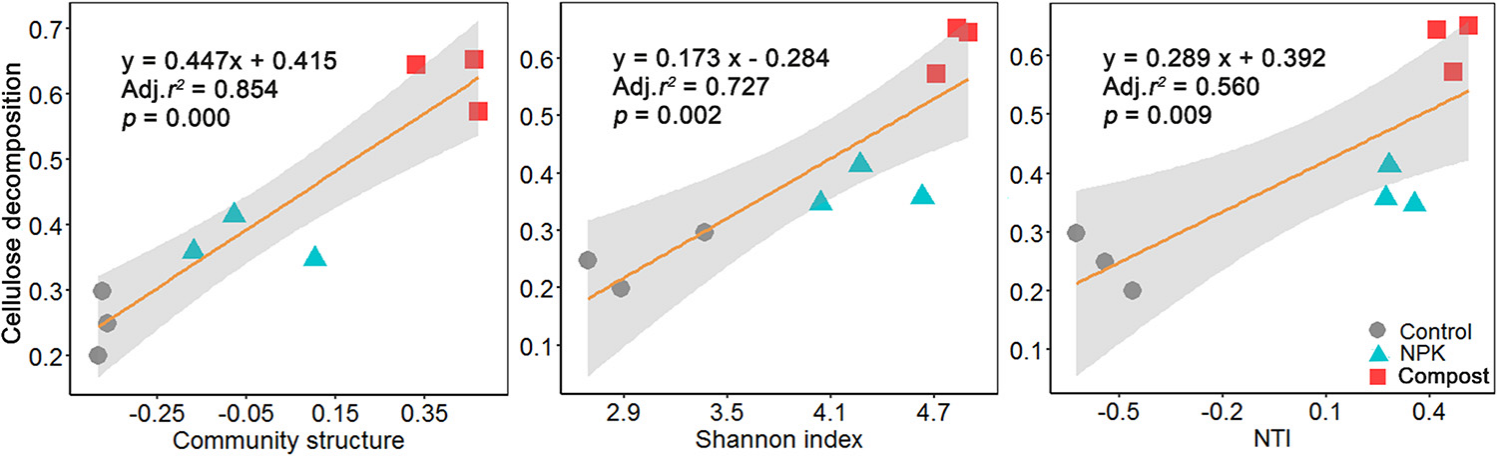
Relationships between cellulose decomposition and the community characteristics of ^13^C-assimilating fungi in soils undergoing long-term fertilization. The shaded area is the 95% confidence interval of the regression line. Cellulose decomposition is expressed as the percentage of cellulose-derived C loss.

Regression analysis revealed that fungal community structure (as represented by the first principal component) was positively (*P < *0.01) correlated with cellulose decomposition rate (Fig. 5). Further, the cellulose decomposition rate also increased as a function of soil fungal diversity (Shannon index) and NTI. These results indicated that ^13^C-assimilating fungal communities had substantial effects on cellulose decomposition.

### Comparison of ^13^C-assimilating fungal community composition and diversity determined by DNA- and RNA-SIP.

The RNA-SIP technique revealed that *Ascomycota* dominated cellulose utilization in all soil treatments (Fig. 3), which is consistent with results of the DNA-SIP technique (Fig. S5). However, these two techniques uncovered different ^13^C-labeled fungal communities in soils. Compared with NPK and unfertilized soils, compost soil increased the relative abundance of *Basidiomycota* at the RNA level while increasing that of *Ascomycota* at the DNA level. Meanwhile, the RNA-SIP technique showed higher levels of fungal diversity across all test soils in comparison with DNA-SIP (Fig. 2 and Fig. S6).

10.1128/mbio.00323-22.5 FIG S5 Relative abundances of major phyla of the ^13^C-assimilating fungal communities (>1%) among the soils undergoing long-term fertilization obtained by the DNA-SIP technique. Download FIG S5, DOCX file, 0.1 MB. Copyright © 2022 Miao et al. 2022 Miao et al. https://creativecommons.org/licenses/by/4.0/ This content is distributed under the terms of the Creative Commons Attribution 4.0 International license.

10.1128/mbio.00323-22.6 FIG S6 Shannon diversity and observed OTUs of ^13^C-assimilating fungal communities in soils undergoing long-term fertilization obtained by the DNA-SIP technique. Different letters indicate significant differences (*P < *0.05) among the fertilization treatments. Download FIG S6, DOCX file, 0.06 MB. Copyright © 2022 Miao et al. 2022 Miao et al. https://creativecommons.org/licenses/by/4.0/ This content is distributed under the terms of the Creative Commons Attribution 4.0 International license.

## DISCUSSION

### Fungal communities regulated by fertilization influence cellulose decomposition.

Long-term compost amendment altered the composition of the ^13^C-assimilating fungal community and strongly influenced soil cellulose C turnover. *Dothideomycetes* dominated cellulose utilization in compost soil, whereas *Sordariomycetes* were more prevalent in both NPK and unfertilized soils (Fig. 3). These results are consistent with those of Schneider et al. (31), who found that *Sordariomycetes* and *Dothideomycetes* (all *Ascomycetes*) were the dominant cellulase producers for cellulose decomposition and reported their key involvement in the breakdown of plant biomass (32, 33). *Dothideomycetes* commonly occur in more extreme ecological niches and exhibit a considerable capacity to maintain cooperative metabolic associations with other species (34). For example, *Dothideomycetes* were associated with the depolymerization of recalcitrant polymers during plant litter decomposition (35) and could serve as indicators for slow and passive organic C decomposition in the upper-layer soil (0- to 15-cm depth) of the Alaskan tundra (36). Accordingly, in compost soil, the input of complex organic materials favored the growth of *Dothideomycetes* (37), thereby contributing to the breakdown of cellulose. In contrast, *Sordariomycetes* are ubiquitous in agricultural soils (38), largely because members of this class are fast-growing species that become quickly abundant there given their high capacities to use labile C resources (39). Our previous study found that compared with NPK and control soils, the oxygen effective diffusion coefficient in compost soil was decreased to 1.30 × 10^−6^ m^2^ s^−1^ from 3.05 × 10^−6^ to 5.19 × 10^−6^ m^2^ s^−1^ due to more macroaggregate formation (40). It is likely that more oxygen availability in NPK and unfertilized soils favors the proliferation of *Sordariomycetes* species, in that most of them are aerobic. Members of *Sordariomycetes* are able to use a wide variety of substrates, and the majority of them are known to have saprotrophic abilities (41). Therefore, they generally flourish in response to cellulose-rich straw amendments and are key decomposers of organic materials in soils (42).

Microbial biomarker analysis can advance the understanding of how microbial communities modulate the decomposition process of organic materials in soils. Here, the genus Cryptococcus, in the phylum *Basidiomycota*, was more abundant in compost soil than NPK and control soils (Fig. 4). Members of Cryptococcus are characterized as oligotrophs and often adapt well to severe environments, such as polar regions (43) and arid soils (44), with the help of polysaccharide capsules, which enable a better access to nutrients via fungal hyphae (45). The unfavorable soil niches in compost soil, like labile C deficiency and low oxygen concentration (37, 40), therefore are beneficial for the proliferation of Cryptococcus. Previous studies documented that Cryptococcus has the potential to improve soil C cycling, inhibit pathogens, and promote crop yield (46). It seems that some members of Cryptococcus improved microbiota activity by suppressing the cytotoxicity of pathogens and accelerated substrate C turnover. Furthermore, Cryptococcus is well known for its high capacity to decompose complex organic substances by producing extracellular enzymes (47) and shows negative correlations with SOC content (48). Consequently, the enhanced population of Cryptococcus potentially increased catabolism rates of cellulose derived C by increasing enzyme production and reduced ^13^C sequestration efficiency.

We found that the NTI values for ^13^C-assimilating fungi were higher than zero in compost soil yet close to zero in NPK and control soils, indicating that ^13^C-labeled fungal communities in compost soil were phylogenetically clustered and had higher species relatedness. Environmental filtering is thought to play a key role in the assembly of fungal communities (49). In this respect, the availability of organic materials has been shown to impose a stringent filter on fungal taxa for the selection of closely related species (50, 51). First, compost amendment typically incorporates narrow-spectrum C resources, such as stable hydrophobic materials and lignocellulose, into soil (37). This would strengthen the niche-filtering effect on the fungal community according to the species-sorting concept (52), since most fungal species have particular preferences for certain substrates (53), resulting in phylogenetic niche conservatism of fungal communities in compost soil (54). Second, biological invasion from added organic fertilizers possibly filtered out some native soil microbial species, whose competitive advantage is low, through strengthened interspecific competition (13). These processes would lead to the extinction of some fungal species due to their poor adaptation to abrupt changes in environmental conditions and, accordingly, reduced fungal diversity (55). However, compost soil harbored higher fungal diversity than NPK and control soils (Fig. 2). The direct input of organic fertilizers introduces diverse fungal species, the majority of which can persistently colonize the soil due to their preference for recalcitrant resources (56, 57). Following a 15-year organic fertilization, Sun et al. (13) found that exogenous fungal species from added manure accounted for up to 10.9% of soil fungal richness. Therefore, the greater diversity we observed in compost-treated soil could have arisen from the introduction of exogenous fungi.

Microbial diversity is pivotal in soil nutrient cycling processes such as C decomposition (58–60). Here, the diversity of ^13^C-labeled fungi was positively correlated with cellulose decomposition rates (Fig. 5). This result is consistent with work by Juarez et al. (61) and Maron et al. (62); using a dilution-to-extinction approach in microcosm experiments, they found that SOC mineralization increased as soil microbial diversity increased. These findings suggest that the coexistence of multiple fungal groups may promote their functional capacities and hasten the C cycling process (63, 64). The complementarity function niche hypothesis states that many distinct species can utilize C resources successively, by producing complementary enzymes during the substrate decomposition process (65). Consequently, fungal communities with higher diversity are more apt to generate greater complementarity effects, which could have contributed to the depolymerization of cellulose in compost soil. Moreover, the observation that the diverse fungal taxa were characterized by pronounced clustering and connectivity in compost soil suggests a strengthened pattern in synergistic interactions for C utilization (66). Microbial groups with a high degree of interspecies dependence can induce more complex and positive interactions, leading to high C consumption in soils with long-term unbalanced fertilization (67). Therefore, it is likely that in our study, the compost amendment increased the capacity of soil fungal species to decompose cellulose-rich substrates by enhancing such complementarity interactions, whose outcome is a better collective exploitation of cellulose-derived C in soil (68).

### Comparison of ^13^C-assimilating fungal communities determined by DNA- and RNA-SIP.

Similar to our previous measurement of ^13^C-assimilating fungal community using DNA-SIP (Fig. S5), the RNA-SIP technique also identified *Ascomycota* dominating cellulose utilization across all soil treatments (Fig. 3). This is because those members of *Ascomycota* (mostly saprotrophic fungi) are highly enriched in arable soils and thrive in response to cellulose amendments (38, 41). However, the RNA-SIP and DNA-SIP techniques uncovered different ^13^C-labeled fungal communities in soils. Compost amendment increased the relative abundance of *Basidiomycota* at the RNA level while increasing that of *Ascomycota* at the DNA level compared with NPK and unfertilized soils.

The RNA-based microbial species are more sensitive to changes in soil resource availability due to their rapid incorporation of substrate-derived C into RNA (69). As such, they are expected to be metabolically active at the time of sampling (30, 70). Another advantage to using RNA-SIP is that it requires a lower substrate ^13^C enrichment of 10 atom% (71) than the 20 atom% needed for DNA-SIP (72). Hence, the RNA-based SIP technique could effectively target slow-growing microbial species capable of actively synthesizing RNA but not DNA. The low oxygen availability in compost soil due to increased macroaggregation possibly suppressed the growth of fast-growing *Ascomycota* (40). The reduced ^13^C content in compost soil during incubation also adversely affected *Ascomycota*’s proliferation, since its members generally tend to thrive on C-rich substrates (42). Conversely, more recalcitrant organic substances derived from cellulose, such as microbial necromass and by-products, were readily available for *Basidiomycota*, whose members are characterized by low growth rates and prefer to decompose recalcitrant polymers (39). In contrast, the DNA-SIP technique may favor fast-growing fungi with high turnover rates that incorporate most of the newly added ^13^C to repair or duplicate their DNA (73). Moreover, the DNA-SIP analysis tends to target the most abundant functional members of a community, including its dead and metabolically active taxa, simply because DNA persists longer than RNA in soil (74). Consequently, compared with RNA-SIP, the DNA-SIP approach is liable to overestimate the relative abundance of metabolically active *Ascomycota*. Our results suggest that RNA-based microbial analysis could be more robust at detecting ecologically active microorganisms, especially slow-growing microbes, in response to variations in available soil resources.

The RNA-SIP technique revealed higher levels of ^13^C-labeled fungal diversity across all test soils in comparison with DNA-SIP (Fig. 2 and Fig. S6), indicating that RNA-SIP could recover fungal diversity more comprehensively than DNA-SIP (75). This is because microorganisms with low isotopic incorporation arising from their low growth rate and low competitive advantage for C resources can be reliably detected by RNA-SIP (70, 71). Interestingly, at the RNA level, the compost soil featured higher fungal diversity than the NPK and control soils, but this pattern was reversed at the DNA level. This suggests that in compost treatment, more diverse species participated in cellulose utilization and fungal synergistic interactions might have played a more important role than expected by DNA-SIP. Therefore, our work emphasizes the importance of using the RNA-SIP technique to discern active participants in substrate utilization and to comprehensively assess microbial contributions to decomposition processes in soils.

### Conclusions.

How long-term application of compost and NPK fertilizers affects soil fungal communities and the consequences for cellulose decomposition were both experimentally investigated in this study. *Dothideomycetes* (mainly the genus Cryptococcus) dominated cellulose utilization in compost soil, whereas the copiotrophic *Sordariomycetes* were more abundant in both NPK and unfertilized soils. The compost amendment promoted fungal diversity and phylogenetic relatedness and strengthened the decomposition capacity of fungi for cellulose-rich substrates by enhancing synergistic interactions. The RNA-based SIP technique is sensitive enough to detect responses of fungi to local shifts in soil resource availability and could efficiently distinguish slow-growing microorganisms. Overall, because of the augmented decomposition capacity of fungal species for cellulose-rich substrates, the accumulation of cellulose-derived C is less efficient in compost-treated soil.

## MATERIALS AND METHODS

### Soil sampling.

The field experiment was established in 1989 at the Fengqiu State Key Agro-ecological Experimental Station (35°00′N, 114°24′E) in Henan Province, China. Soil in the study region was derived from alluvial sediments of the Yellow River and classified as an Aquic Inceptisol (76). The experimental field site had been developed for a cropping rotation system of winter wheat (Triticum aestivum) followed by summer maize (Zea mays), for which detailed information can be found in the work by Miao et al. (77). Soil samples (0- to 20-cm depth) were collected from three treatments: no fertilizer (control), nitrogen-phosphorus-potassium fertilizer (NPK), and compost. Each treatment had four replicate plots based on a completely randomized design, and soil samples from each plot were mixed to form a composite. Each soil sample was divided into two subsamples: one was stored at 4°C for the SIP incubation, and the other was air dried for analysis of soil properties (Fig. S1).

### Microcosm experiment.

For each treatment soil, three groups were established: (i) soil with [^12^C]cellulose added; (ii) soil with [^13^C]cellulose added; and (iii) soil without cellulose. Fresh soil samples (each 10 g, on an oven-dried basis) were placed in 100-mL incubation jars. The ^13^C-labeled cellulose (2 mg g^−1^; 97 atom% ^13^C; produced from maize [Zea mays] straw; uniformly labeled; IsoLife, Wageningen, the Netherlands) and ^12^C cellulose (1.93 mg g^−1^; <1.2 atom% ^13^C) were added to the soil and immediately homogenized. Soil water-holding capacity was maintained at 60% by adding deionized water, every other day, using a minipipette. The top of each jar was covered by a plastic wrap with needle-punctured holes to maintain aerobic conditions; all jars were incubated at 20°C in the dark. Three replicates per group were destructively sampled 20 days later for microbial analysis and determinations of δ^13^C values and content of organic C. The SOC content was quantified using a wet oxidation-redox titration. To measure δ^13^C, soil samples were pretreated with HCl to remove any inorganic C and then analyzed using a MAT 253 isotope ratio mass spectrometer (Thermo Electron, Bremen, Germany).

### RNA extraction and stable isotope probe gradient fractionation.

Total RNA was extracted from fresh soil per sample, using the RNA power soil isolation kit (MO BIO Laboratories, CA, USA), with DNase I used to remove any contaminant DNA from the extracted RNA. These RNA samples were purified further using the RNeasy minikit (Qiagen, Hilden, Germany), after which quality and quantity of purified RNA were checked with a NanoDrop 1000 spectrophotometer (Wilmington, DE, USA). Next, ca. 500 ng of this purified RNA was mixed with a cesium trifluoroacetate (CsTFA) gradient buffer (0.1 M Tris-HCl, pH 8.0; 0.1 M KCl; 1 mM EDTA), to achieve a buoyant density of 1.790 g mL^−1^. Each sample mixture was spun in a VTI 65.2 vertical rotor (Beckmann Coulter Inc., USA) using an Optima XPN 80 centrifuge (Beckman Coulter Inc., USA), at 130,000 × *g* for 65 h at 20°C. The ensuing centrifuged RNA gradients were then fractionated using a peristaltic pump (ISM850; Ismatec, Switzerland) and the buoyant density of each fraction was measured by an AR200 digital refractometer (Reichert, USA).

For RNA precipitation, all fractions were mixed with isopropanol, and RNA pellets were air dried and resuspended in 20 μL of RNase-free sterile water. The cDNA for each fraction was synthesized using the total RNA as a template, according to the manufacturer’s instructions provided with HiScript II reverse transcription SuperMix (Bio-Rad, CA, USA). Copy numbers of the fungal internal transcribed spaced (ITS) gene in each fraction were determined by quantitative PCR (qPCR), using the primer set ITS1F-ITS2 (78) with synthesized cDNA as the template, in a Bio-Rad S1000 machine (Bio-Rad Laboratories, CA, USA). The thermal cycle protocol was as follows: 95°C for 3 min followed by 40 cycles of 95°C for 30 s, 55°C for 30 s, and 72°C for 30 s, with a 10-min final extension at 72°C. A standard curve was derived using a serial 10-fold dilution of plasmids harboring the ITS gene. Every amplification yielded a single peak and the amplification efficiencies of our assays were 91.0 to 96.5%, with high coefficients of determination (*r*^2^ = 0.961 to 0.998).

### Illumina HiSeq sequencing and bioinformatics analysis.

We chose RNA samples from the [^13^C]cellulose microcosms and the corresponding fractions from [^12^C]cellulose microcosms at high density for further analysis. These RNA samples were reverse transcribed into cDNA for their Illumina amplicon sequencing. For this, the same primer sets were used as for the ITS gene amplification described above. The PCR products were purified using an EZNA gel extraction kit (Omega, USA). Then, to yield the sequencing libraries, the NEBNext Ultra DNA library preparation kit was used according to the manufacturer’s instructions. High-throughput sequencing was performed on an Illumina HiSeq 2500 platform (Illumina, San Diego, CA, USA) to generate 250-bp paired-end reads. The assembly of these paired-end reads was done using the FLASH tool (79), with the Quantitative Insights into Microbial Ecology (QIIME) pipeline (80) used to perform the quality filtering of reads. The resulting high-quality sequences were then clustered into operational taxonomic units (OTUs) at a 97% similarity by the UPARSE algorithm (81). Representative sequences, those most abundant per OTU, were taxonomically annotated with the RDP classifier (34). To determine differences between samples, a randomly selected subset of 108,055 sequences per sample underwent a downstream analysis.

### Statistical analyses.

Significant differences in the proportions of cellulose-derived ^13^C, the Shannon diversity index, observed OTUs, and the NTI values for the three fertilization treatments were determined by one-way analysis of variance (ANOVA), followed by a least-significant-difference (LSD) test at a *P* value of <0.05, in SPSS 19.0 for Windows (IBM Corp., Armonk, NY, USA). Both a principal-coordinate analysis (PCoA) and hierarchical clustering with unweighted pair group method with arithmetic mean (UPGMA) were carried out for fungal communities according to their relative abundance matrix based on Bray-Curtis distances, using the “vegan” and “stats” packages for R (v4.0.3), respectively. Significantly different biomarkers at the genus level were identified using Welch’s *t* test (*P < *0.05) in statistical analysis of metagenomic profiles (STAMP) (82). Relationships between cellulose decomposition and fungal community characteristics were assessed using linear regression models.

To estimate the phylogenetic community structure of soil fungi, the NTI was calculated for each sample using the *ses.mntd* function in the “picante” package for R (83). An NTI value significantly greater than zero indicates that coexisting species have closer associations than expected by chance (phylogenetic clustering). Conversely, an NTI significantly less than zero indicates that the species have more distant associations than expected by chance (i.e., phylogenetic overdispersion) (49). For these analyses, a phylogenetic tree based on aligned representative sequences was constructed in MEGA 7.0 software.

### Data availability.

The raw sequence data were submitted to the NCBI Sequence Read Archive (SRA) with accession number PRJNA774483.
