# Correction for Miao et al., “Long-Term Compost Amendment Spurs Cellulose Decomposition by Driving Shifts in Fungal Community Composition and Promoting Fungal Diversity and Phylogenetic Relatedness”

**DOI:** 10.1128/mbio.02956-22

**Published:** 2022-11-03

**Authors:** Yuncai Miao, Junjie Li, Ye Li, Yuhui Niu, Tiehu He, Deyan Liu, Weixin Ding

## AUTHOR CORRECTION

Volume 13, no. 3, e00323-22, 2022, https://doi.org/10.1128/mBio.00323-22. Page 1, abstract, lines 8 and 9: “(mainly members of the genus *Cryptococcus*)” should be deleted.

Page 9, line 10: “(mainly the genus *Cryptococcus*)” should be deleted.

