# Supplementary material for: Long-Term Compost Amendment Spurs Cellulose Decomposition by Driving Shifts in Fungal Community Composition and Promoting Fungal Diversity and Phylogenetic Relatedness
Source: mBio. 2022 May 2;13(3):e00323-22. doi: 10.1128/mbio.00323-22 (PMC9239258; doi:10.1128/mbio.00323-22)

**Fig. S1.** Basal soil characteristics after 27 years of fertilization. The whiskers denote standard errors of the means (*n* = 3). Differing letters indicate significant differences among the fertilization treatments, at *p* < 0.05.


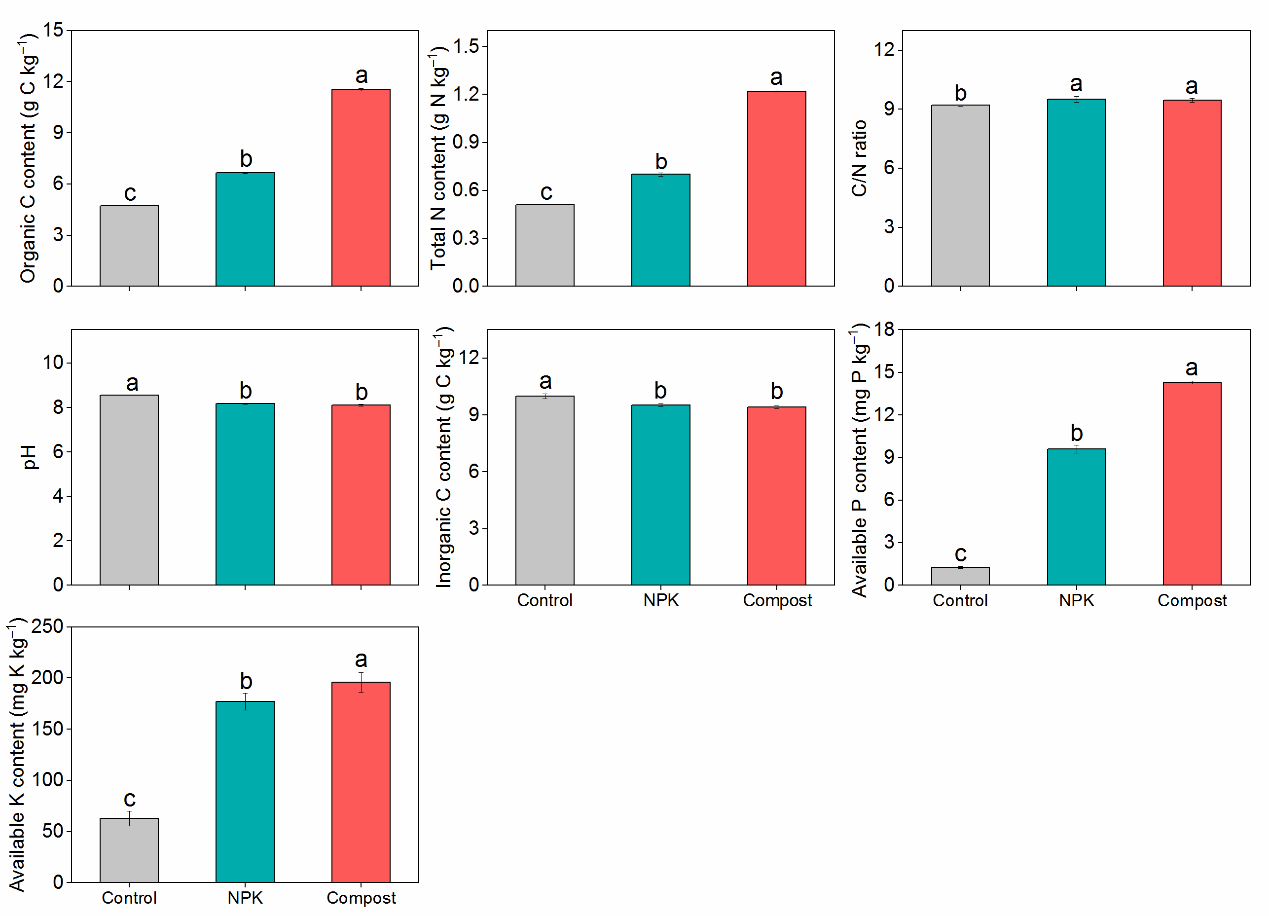

Supplement: FIG S1 [file mbio.00323-22-s0001.docx]
