# Supplementary material for: Long-Term Compost Amendment Spurs Cellulose Decomposition by Driving Shifts in Fungal Community Composition and Promoting Fungal Diversity and Phylogenetic Relatedness
Source: mBio. 2022 May 2;13(3):e00323-22. doi: 10.1128/mbio.00323-22 (PMC9239258; doi:10.1128/mbio.00323-22)

**Fig. S3.** Relative abundances of fungal ITS-RNA transcripts, based on the CsTFA buoyant density, at 20 days of incubation with ^12^C- (blue) and ^13^C-cellulose (red). The amounts of ITS-RNA transcripts were determined by qPCR, for which cDNA served as the template.


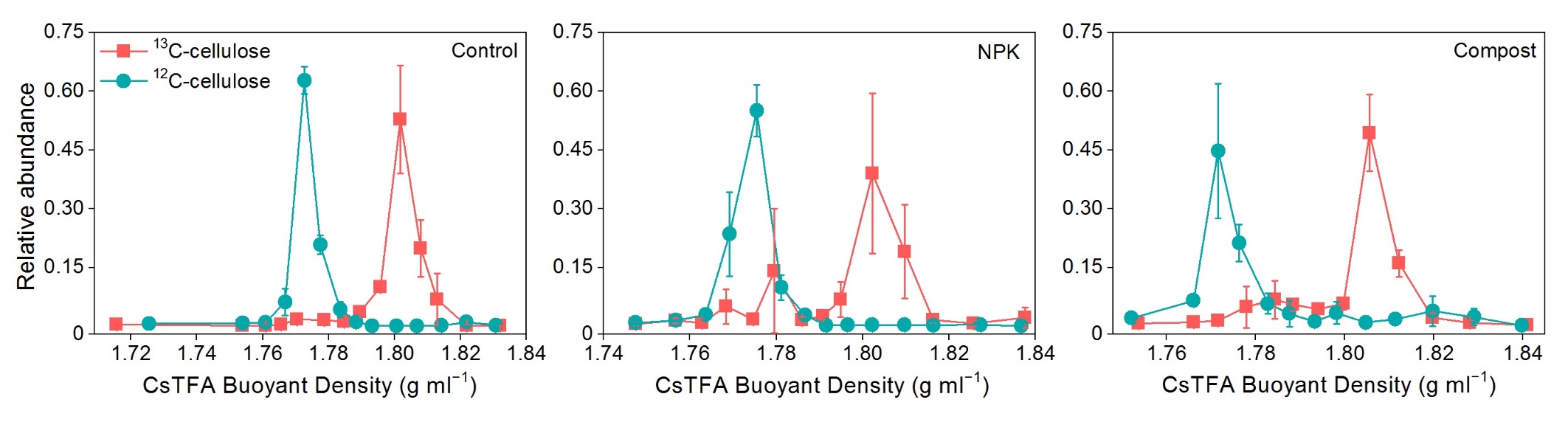

Supplement: FIG S3 [file mbio.00323-22-s0003.docx]
