# Supplementary material for: Long-Term Compost Amendment Spurs Cellulose Decomposition by Driving Shifts in Fungal Community Composition and Promoting Fungal Diversity and Phylogenetic Relatedness
Source: mBio. 2022 May 2;13(3):e00323-22. doi: 10.1128/mbio.00323-22 (PMC9239258; doi:10.1128/mbio.00323-22)

**Fig. S4.** Principal coordinates analysis (PCoA) of fungal communities in the heavy fractions across the fertilization treatments receiving the ^12^C- and ^13^C-cellulose additions, based on Bray-Curtis distances at the operational taxonomic unit (OTU) level, at 20 days of incubation.


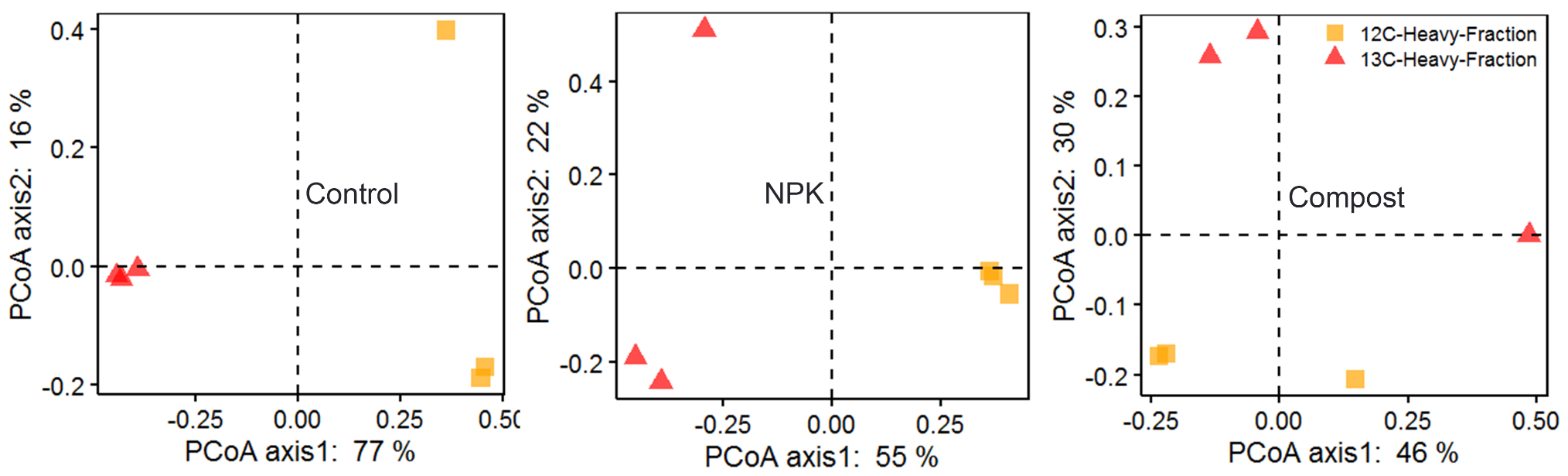

Supplement: FIG S4 [file mbio.00323-22-s0004.docx]
