# Supplementary material for: Long-Term Compost Amendment Spurs Cellulose Decomposition by Driving Shifts in Fungal Community Composition and Promoting Fungal Diversity and Phylogenetic Relatedness
Source: mBio. 2022 May 2;13(3):e00323-22. doi: 10.1128/mbio.00323-22 (PMC9239258; doi:10.1128/mbio.00323-22)
Supplement: FIG S5 [file mbio.00323-22-s0005.docx]

**Fig. S5.** Relative abundances of major phyla of the ^13^C-assimilating fungal communities (> 1%) among the soils under long-term fertilization obtained by the DNA-SIP technique.

**
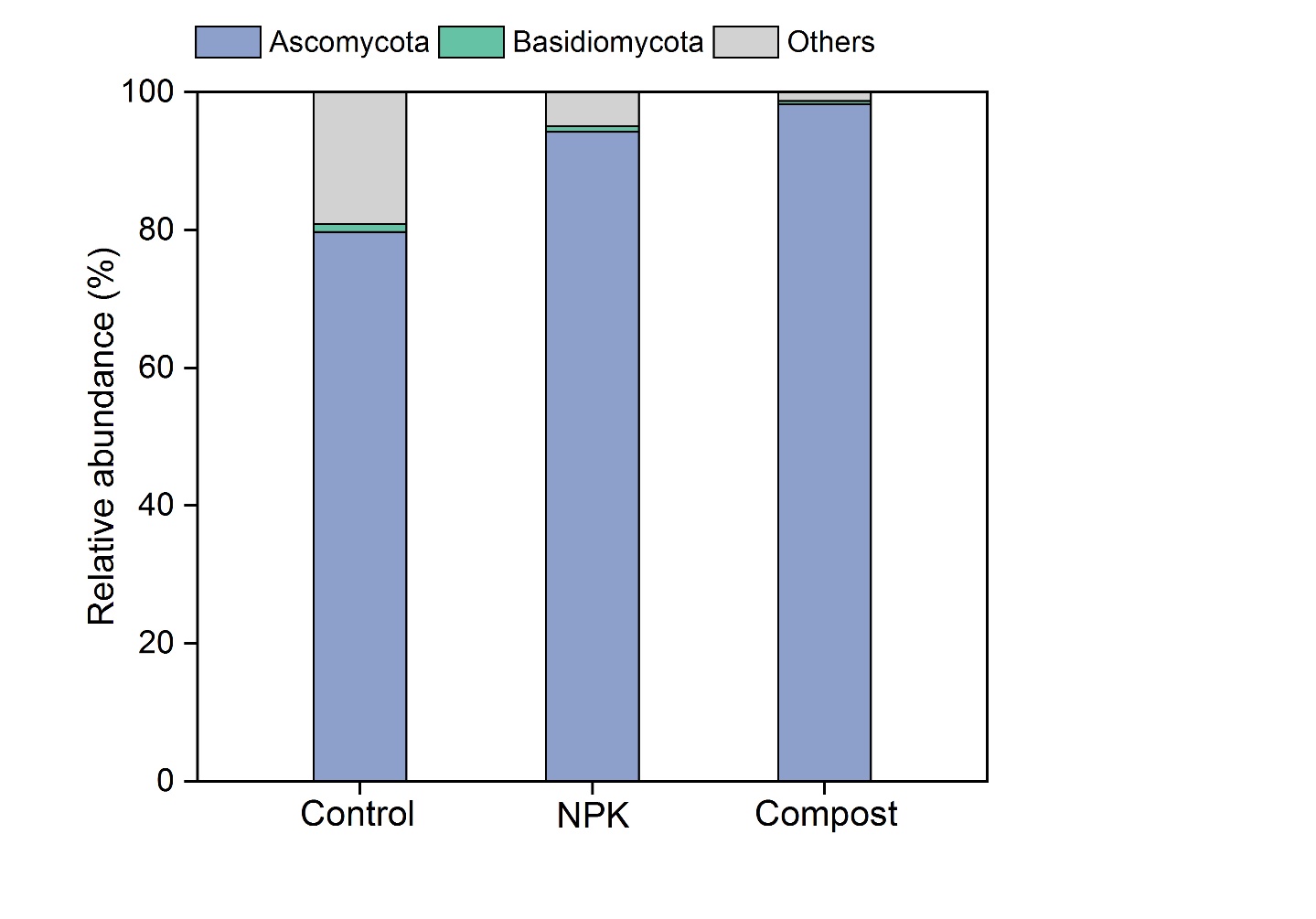
**
