# Supplementary material for: Long-Term Compost Amendment Spurs Cellulose Decomposition by Driving Shifts in Fungal Community Composition and Promoting Fungal Diversity and Phylogenetic Relatedness
Source: mBio. 2022 May 2;13(3):e00323-22. doi: 10.1128/mbio.00323-22 (PMC9239258; doi:10.1128/mbio.00323-22)

**Fig. S6.** Shannon diversity and observed OTUs of ^13^C-assimilating fungal communities in soils under long-term fertilization obtained by the DNA-SIP technique. Differing letters indicate significant differences (*p* < 0.05) among the fertilization treatments.


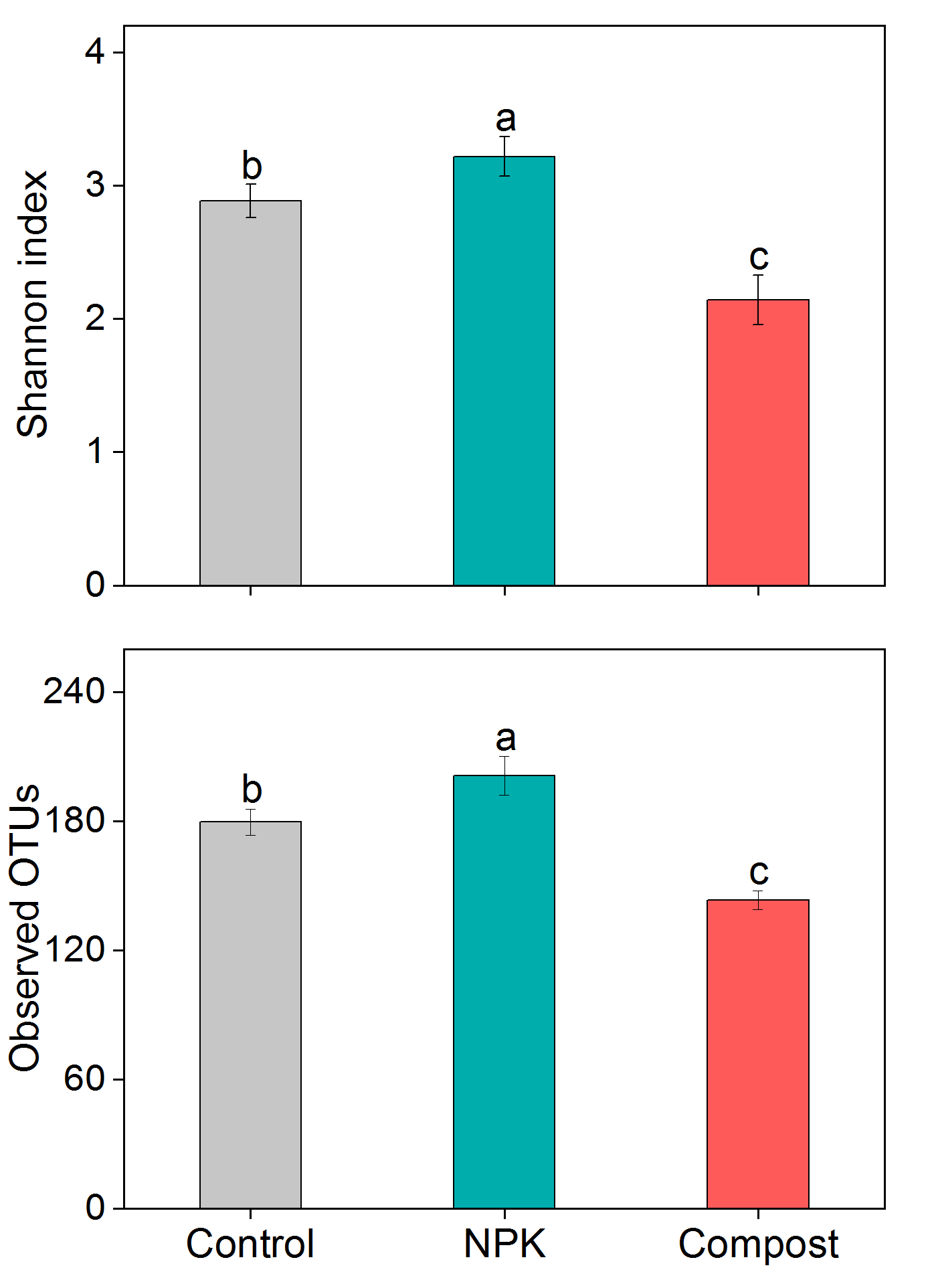

Supplement: FIG S6 [file mbio.00323-22-s0006.docx]
